# Primary intracranial rhabdomyosarcoma: A systematic review of existing literature from 2000 to 2024

**DOI:** 10.12669/pjms.41.13(PINS-NNOS).13473

**Published:** 2025-12

**Authors:** Haseeb Mehmood Qadri, Momina Khawaja, Arham Amir Khawaja, Haseeb Ahmad, Momin Bashir, Syed Shahzad Hussain Shah, Asif Bashir

**Affiliations:** 1 Haseeb Mehmood Qadri, MBBS. Postgraduate Resident, Neurosurgery, Punjab Institute of Neurosciences, Lahore, Punjab, Pakistan; 2 Momina Khawaja, MBBS. Medical Graduate, Gulf Medical University, Ajman, UAE; 3 Arham Amir Khawaja, MBBS. Postgraduate Resident, General Surgery and Surgical Oncology, Shaikh Zayed Medical Complex, Lahore, Pakistan; 4 Haseeb Ahmad, Medical Student, Ameer Ud Din Medical College, Lahore, Pakistan; 5 Momin Bashir, Medical Student, New York University, USA; 6 Syed Shahzad Hussain Shah, MBBS, FCPS.Professor of Neurosurgery, Unit-II, Punjab Institute of Neurosciences, Lahore, Punjab, Pakistan; 7 Asif Bashir, MBBS, MD, FAANS, FACS Professor of Neurosurgery, Unit-I, Punjab Institute of Neurosciences, Lahore, Punjab, Pakistan

**Keywords:** Rhabdomyosarcoma, Brain Neoplasms, Vincristine, Headache, Facial Paralysis, Vomiting, Hydrocephalus

## Abstract

**Background and Objective:**

Primary Intracranial Rhabdomyosarcoma (PIRMS) represents a rare and poorly understood entity within brain tumours. The objective was to evaluate the clinicoradiological presentation and optimal management strategies for PIRMS.

**Methodology:**

A comprehensive literature search was conducted in PubMed Central and Google Scholar from 2000 to 2024. Only English-language, open-access articles with confirmed human PIRMS cases were included. Quality was assessed using Joanna Briggs tools, and data synthesis followed PRISMA guidelines, registered with PROSPERO with registration number CRD42024507092. A total of 176 articles were screened, and finally, 27 were included, comprising data on 39 patients.

**Result:**

About 22 (56.41%) patients were adults, and 17 (46.59%) were pediatric, with a male predominance of 23 cases (58.67%). Most patients (25) were Asians (64.1%). Headache, facial nerve palsy, and nausea/vomiting in 58.97% (23), 28.21% (11) and 25.64% (10) were the most common findings. Imaging showed ventriculomegaly and haemorrhage, in 10.26% (4) each on computed tomography, and hyperintensities on T1WI in 20 cases (51.28%). About 17.95% (7) of lesions were in the pineal region. Gross-total resection was performed in 15 cases (38.46%). On histopathology, rhabdomyoblasts and round cells were found in 25.64% (10) and 38.46% (15). The mean follow-up of 28 patients was 14.39 +- 17.68 months.

**Conclusion:**

Despite limitations, PIRMS remains difficult to diagnose preoperatively, with histopathology as the gold standard. Treatment response varies, but adjuvant chemoradiotherapy and gross total resection improve outcome.

## INTRODUCTION

Rhabdomyosarcoma (RMS) comprise an aggressive malignant tumour of mesenchymal origin, arising in 40% of cases from the head and neck region, classically divided into orbital, parameningeal, and non-parameningeal.^1^ This tumour is exceedingly rare in the adult population and mostly affects pediatric patients, with less than one per cent of malignancies in adults and eight per cent of solid tumours in children.^2^ Despite recent advances, the prognosis of the disease has been dismal. The main reasons for poor prognosis in adults are unfavourable primary sites and higher rates of regional and distant spread, histological type (pleomorphic has worse outcome), and genetics (chromosomal translocations, fusion of two transcription factor-encoding genes (PAX3/7 and the FOXO1) which translate into metastatic presentation, recurrence and resistance to current regimes.^3^

The primary therapy of tumours consists of a multidisciplinary approach using multiagent chemotherapy and local control with surgical resection and/or ionising radiation.^4^ There is ambiguity of the oncological origin of the tumour in the brain parenchyma and the genetic element involved. Unfortunately, there is not much literature available to identify key points in diagnosis and management when RMS involves the brain parenchyma. Therefore, PIRMS management is a challenge for medical, radiation oncologists and neurosurgeons.

We aimed to conduct a systematic review of all available publications on the PIRMS. Through this review, we aimed to stratify the findings and consolidate them, leading to earlier diagnosis and optimising management of such rare, aggressive tumours when they involve the brain parenchyma. Although a systematic review on PIRMS exists, it primarily incorporates older cases. An updated review is warranted to include the most recent reports, following a thorough appraisal of existing literature, as part of a continued effort to deepen our understanding of this rare pathology.

## METHODOLOGY

**Fig.1:**
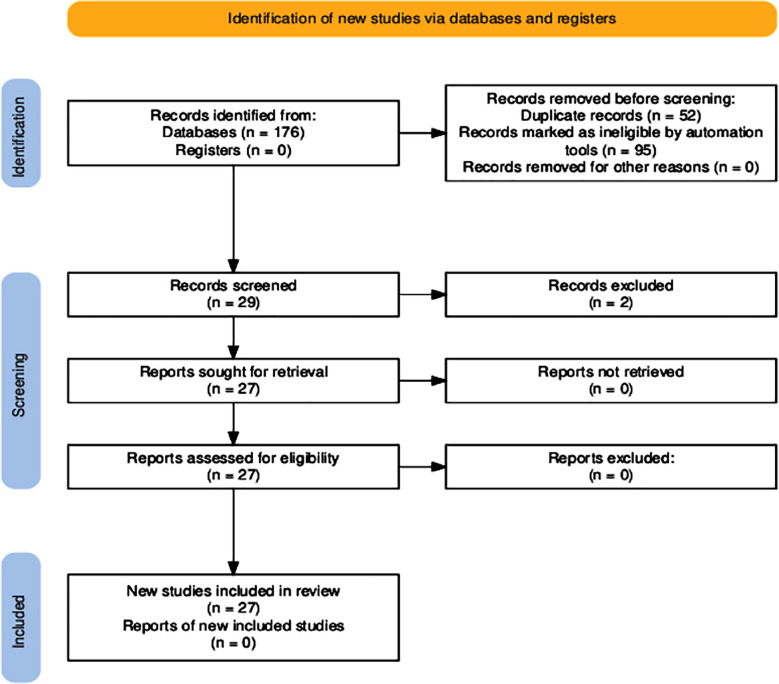
Preferred Reporting Items for Systematic Reviews and Meta-Analyses (PRISMA) flowchart for search strategy and the quality assessment of included studies.^5^

A systematic review of the existing literature was conducted between 1st January 2000 and 31st December 2024, incorporating all diagnosed cases of primary intracranial rhabdomyosarcoma. The Preferred Reporting Items for Systematic Reviews and Meta-Analysis (PRISMA) strategy^5^ was used to stratify the articles (Fig.1). The review is registered with PROSPERO with registration number CRD42024507092.

An extensive literature search was conducted from PubMed Central and Google Scholar using predefined keywords to isolate all of the published articles. Booleans operators (AND/OR) were used to formulate keywords combinations which are listed as follows: ‘Rhabdomyosarcoma Brain’, ‘Primary Rhabdomyosarcoma Brain’, ‘Rhabdomyosarcoma AND Brain’, ‘Primary Rhabdomyosarcoma AND Brain’, ‘Primary Intracranial Rhabdomyosarcoma’, Primary Intraparenchymal Rhabdomyosarcoma Brain’, ‘Primary Brain Rhabdomyosarcoma’. These keyword combinations were used to search all the publications on the topic of discussion. The articles were selected based on the predefined inclusion and exclusion criteria

### Inclusion criteria

All case reports, case series, and original articles which were confirmed cases of primary intracranial rhabdomyosarcoma (on whole body imaging and histopathology) of living human cases were selected. Rhabdomyosarcomas were proven upon histopathology, and primary lesions in the brain were confirmed by whole-body imaging. Only articles in the English language or non-English articles with translated versions available and open access were recruited.

### Exclusion criteria

All animal and cadaveric studies, review articles, short communications, letter to editor, editorials, pictorial essays, and conference abstracts were excluded. In addition, all articles that had incomplete information were also excluded.

### Data collection and analysis:

Literature search was conducted primarily by HMQ. The data was collected by four independent data collectors, HMQ, AAK, MB, and HA. The data from the selected articles was entered on a premade data collection form on Microsoft Word 365 and Microsoft Excel 365 (Redmond, WA: Microsoft Corp.). Both qualitative and quantitative variables were included; demographic details (age and gender), clinical manifestations, radiological findings, treatment modalities and surgical approaches, histopathological findings, complications, prognosis and follow-up details were documented. All extracted data were imported into Statistical Package for the Social Sciences software version 24 (Armonk, NY: IBM Corp.) for statistical analysis. Descriptive statistics were used to summarise the data. Tables were generated to illustrate the findings.

Using the initial keyword search on selected databases, a total of 176 articles were isolated. A total of 52 articles were removed due to duplications, and 95 articles were removed by the automation tool. A total of 29 articles underwent initial screening using Joanna Bridge critical appraisal tools (JBI) (see appendix, Tables-VII, VIII and IX). Two articles were excluded using the JBI assessment. All 27 articles’ reports were retrieved. Finally, a total of 27 articles were included, consisting of 25 case reports and one case series and one original article, containing data on 39 patients (Fig.1). The details of all included studies are summarized in Table-I.

**Table-I:** Summary of all previously reported cases of PIRMS, including surgical approach, neoadjuvant or adjuvant therapy, and clinical outcomes.

Study by	Cases	Neo-adjuvant therapy	Type of management (surgery or adjuvant therapy or both)	Surgical approach	Adjuvant therapy	Outcome/survival
Mondrago et al.^6^	1	Nil	Both	Transcortical transventricular	temozolomide RT- (63 Gy)	Death- 21 months
Sakaguchi et al.^7^	1	Nil	Both	Nil	doxorubicin, etoposide, cisplatin RT (50.4 Gy)	Survived- 68 months follow-up
Sakaguchi et al.^7^	1	Nil	Both	Craniotomy	vincristine, actinomycin D, Cyclophosphamide RT- 32.4 Gy (focal), 23.4 Gy (craniospinal)	Survived- 11 months follow-up
Vaidya et al.^8^	1	Nil	Surgery	Midline suboccipital craniectomy	Nil	Not specified
Yoshida et al.^9^	1	Nil	Both	Lateral suboccipital craniotomy	Unspecified chemoradiotherapy	Survived- 2 months follow-up
Duncan et al.^10^	1	Nil	Both	Endoscopic transsphenoidal and Craniotomy	vincristine, dactinomycin, cyclophosphamide radiotherapy	Death- 3 months following last resection
Ishi et al.^11^	1	Nil	Both	Left occipital transtentorial	etoposide, cyclophosphamide, cisplatin, ifosfamide, vincristine, pirarubicin, melphalan, carboplatin RT- (focal 22.8 Gy) craniospinal (25.2 Gy)	Survived- 30 months follow-up
Lau et al.^12^	1	Nil	Both	Stereotactic craniotomy using a supracerebellar infratentorial approach	vincristine, dactinomycin, cyclophosphamide	Death- 5 months
Nair et al.^13^	1	Nil	Both	Right retromastoid craniectomy	RT - 5400cGY	Survived – 6 months follow-up
Scull et al.^14^	1	Nil	Surgery	Nil	Not given	Death – 4 months
Palta et al.^15^	1	Nil	Both	Nil	RT - VMAT total dose 5940 cGy vincristine, actinomycin D, cyclophosphamide	Survived- Not specified
Pirillo et al.^16^	1	Nil	Both	Left parietal craniotomy	RT - 60 Gy	Death- 20 months
Carlson et al.^17^	1	Nil	Surgery	Nil	Nil	Death – 3 months
Lee et al.^18^	1	Nil	Both	Bifrontal craniotomy	RT - 45 Gy ifosfamide, carboplatin, etoposide	Survived – 13 months follow-up
Grebe et al.^19^	1	Nil	Both	Nil	RT - 60 Gy	Survived- 11 months follow-up before worsening
Guilcher et al.^20^	1	Nil	Both	Nil	RT - 4500 cGy vincristine, Ifosfamide, carboplatin, etoposide	Survived- 26 months follow-up
Arita et al.^21^	1	Nil	Both	transsphenoidal	RT - 55 Gy Ifosfamide, etoposide, vincristine	Survived- 4 months follow-up
Mahmasani et al.^22^	1	Nil	Both	Nil	RT Vincristine Doxorubicin Cyclophosphamide	Improved
Nishikawa et al.^23^	1	Vincristine, Actinomycin D, Cyclophosphamide, Doxorubicin, Ifosfamide, Etoposide Radiotherapy (20 fractions)	Palliation	Nil	Nil	Death - 6 months
Pandey et al.^24^	1	Nil	Both	Endoscopic third ventriculostomy, infra-tentorial supracerebellar approach.	Vincristine Actinomycin D Cyclophosphamide (VAC)	Death
Zheng et al.^25^	12	Nil	All had surgery, five had adjuvant radiation, three had adjuvant chemotherapy	Nil	Radiation (5, 45.5%) Chemotherapy (3, 27.3%)	8 Death - Mean of 13.7 months follow-up
Masoudi et al.^26^	1	Nil	Both	Right retrosigmoid craniectomy	Chemotherapy	Improved - 6 months follow-up
Jour et al.^27^	1	Nil	Both	Nil	Nil	Improved - 14 months follow-up
De Leeuw et al.^28^	1	Nil	Both	Right frontal craniotomy	Focal radiation (6,000 cGy in 30 fractions). Vincristine weekly Temozolomide daily 4 months of everolimus daily	Death - after 3 years
Desai et al.^29^	1	External beam radiation therapy	Nil	Nil	Nil	Death
Garvia et al.^30^	1	Vincristine, actinomycin, Cyclophosphamide, ifosfamide, Carboplatin, Etoposide (VAC and VICE) Radiotherapy	Both	Nil	Radiotherapy followed by chemotherapy with Vincristine, Actinomycin, Cyclophosphamide and Vincristine, Ifosfamide, Carboplatin, and Etoposide	Death
Zhong et al.^31^	1	Nil	Surgery	Transfrontal	Nil	Death - after 1 month
Xie et al.^32^	1	Six Cycle Temozolomide	Both	Endoscopic assisted pineal mass resection	Nil	Death - after 8 months

## RESULTS

Among the 27 articles comprising 39 patients, the racial distribution was 25 Asian, 11 Caucasian, 2 Hispanic, and one African-American. About 22 (56.41%) of the patients were adults, and 17 (46.59%) were pediatric. About 23 (58.97%) of the patients were male, and 16 (41.02%) were female. The mean age of the adult group was 35.69 years, and the pediatric group was 7.56 years, with a standard deviation of 11.37 and 3.17 years, respectively. The cases were reported in a variety of countries, with the most common being the United States with about nine (33.33%), Japan with six (22.22%) studies. Both China and India contributed about three (11.11%) studies respectively. Mexico, Germany, Korea, Italy, Iran and Spain contributed one (3.70%) study each to the total number (Table-I).

There were a variety of clinical manifestations, with the most common being headache in 23 cases (58.97%), nausea/vomiting in 11 cases (28.21%), facial nerve paralysis in 10 cases (25.64%) and limb weakness in nine cases (23.08%) (Table-II). There were a variety of CT findings, with the most common being ventriculomegaly and haemorrhage, in 10.26% (4) each, respectively (Table-III).

**Table-II:** The frequency and percentage of clinical manifestations of patients

Clinical Manifestations	Number of Cases, n (N=39)	Percentage Occurrence, n/N
Headache	23	58.97%
Facial nerve palsy/weakness	11	28.21%
Nausea/vomiting	10	25.64%
Limb weakness	9	23.08%
Double vision/visual disturbance	5	12.82%
CN VI nerve palsy	5	12.82%
Abnormal gait	4	10.26%
CN III Palsy	4	10.26%
Visual changes	3	7.69%
Fits	3	7.69%
Dysequilibrium	3	7.69%
CN VIII Palsy	3	7.69%
Aphasia	2	5.13%
Hearing loss	2	5.13%
Loss of consciousness	2	5.13%
Papilledema	2	5.13%
Impaired Gag reflex	2	5.13%
Personality changes	2	5.13%
Sensory disturbance	2	5.13%
CN XII	2	5.13%
Others**	11	28.21%

** Neck pain, difficulty swallowing, breathing irregularities, ocular ptosis, tremors, abnormal verbal response, difficulty speaking, sluggish pupillary response, fatigue, cranial nerve I palsy, cranial nerve IX palsy.

**Table-III:** The frequency of common findings on CT scan with percentage occurrence, data isolated from 15 reported cases

Common Findings on CT	Number of Cases, n (N= 39)	Percentage Occurrence (n/N)
Ventriculomegaly	4	10.26%
Hemorrhage	4	10.26%
Edema	2	5.13%
Cystic component	2	5.13%
Midline shift	2	5.13%
Heterogeneous lesion	1	2.56%
Calcified mass	1	2.56%
Mixed density	1	2.56%
Herniation	1	2.56%
High-density lesion	1	2.56%
Solid component	1	2.56%
Ventricular rupture	1	2.56%

There were a variety of MRI findings, with the most common being hyperintense mass on T2WI and hyperintense mass on T1WI, each present in 20 (51.28%) and 16 (41.03%) cases. Enhancement was seen in 10 (25.64%) cases (Table-IV).

**Table-IV:** The frequency of common findings on MRI, with percentage occurrence

Common Findings on MRI	Number of Cases, n (N= 39)	Percentage Occurrence, n/N
Hypointense mass on T1WI	20	51.28%
Hyperintense mass on T2WI	16	41.03%
Enhancement	10	25.64%
Mass effect	6	15.38%
Cystic component	6	15.38%
Heterogeneous lesion	6	15.38%
Edema	5	12.82%
Midline shift	4	10.26%
Ventriculomegaly	3	7.69%
Invasion of parenchyma	2	5.13%
Invasion of vessels	2	5.13%
Isointense on T1WI	2	5.13%
Homogenous mass	2	5.13%
Obstructive hydrocephalus	2	5.13%
Solid component	2	5.13%
Hemorrhage	2	5.13%
Isointense on T2WI	1	2.56%
Hemorrhagic necrosis	1	2.56%
Hypointense lesion on T2WI	1	2.56%
Lobulated	1	2.56%
Herniation	1	2.56%
Effacement	1	2.44%
Fluid Attenuation	1	2.44%
Intraventricular lesion	1	2.44%

Of the lesions, 13 were right-sided (33.33%), 11 were midline (28.21%), 11 were left-sided (28.21%), and two were bilateral (5.13%). There was one case with multiple foci in the brain and spinal cord (2.56%) and another case (2.56%) with multiple foci in the brain. The sites of lesion were organized into three categories: parenchymal, meninges, angles, cisterns and ventricles. The most common sites were pineal in seven cases (16.28%), frontal in five cases (11.63%), and parietal in four cases (9.30%) (Table 5). Sellar region/parasellar/supra-sellar and cerebropontine angle was involved in five (11.63%) and four (9.30%) cases, respectively (Table-V).

**Table-V:** The site of lesions presumably the tumor epifocus and their percentage occurrence

Site of Lesion	Number of Lesions, n (N= 43)	Percentage Occurrence, n/N
***Parenchymal***		
Pineal	7	16.28%
Frontal	5	11.63%
Parietal	4	9.30%
Cerebellum	2	4.65%
Anterior fossa	2	4.65%
Genu of Corpus Callosum	1	2.33%
Parieto-occipital	1	2.33%
Fronto-temporal	1	2.33%
Pituitary	1	2.33%
Occipital	1	2.33%
Temporal	1	2.33%
Petrous temporal	1	2.33%
***Meninges***		
Fronto-parietal meningeal	1	2.33%
***Angles. Cisterns, Ventricles***		
Sellar region/Parasellar/Supra sellar	5	11.63%
Cerebellopontine Angle	4	9.30%
Clival	2	4.65%
Inferior medullary, Cerebello-medullary cistern	1	2.33%
3rd Ventricle	1	2.33%
***Multifocal***		
Multifocal (Brain and/or spinal cord)	2	4.65%

There were a variety of surgical approaches, with the most common being unspecified approaches in eight cases (20.51%), followed by endoscopic transsphenoidal in two cases (5.13%). Other approaches included suboccipital craniotomy/craniectomy, transtentorial, supercerebellar infratentorial approach, retromastoid craniectomy, transfrontal, bifrontal, parietal craniotomies, endoscopic third ventriculostomy, and retrosigmoid craniectomy. Some did not mention approaches specifically.

Among the extents of tumour resection, gross total resection occurred in 15 cases (38.46%), subtotal resection occurred in 16 cases (41.02%), near-total resection occurred in two cases (5.13%), and six cases were unspecified (15.4%).

The histopathological findings were organised into five categories: cell type/morphology, cytoplasm, nucleus, special cells, and patterns. The most common histopathology was rhabdomyoblast/skeletal muscle differentiation in 10 cases (25.64%), and round cells in 15 cases (38.46%) (Table-VI).

**Table-VI:** Microscopic findings of resected specimens, including cell morphology, cytoplasmic and nuclear features, special cell types, and histological patterns.

Histopathology	Number of Cases, n (N= 39)	Percentage Occurrence, n/N
Cell Type/Morphology		
Round cells	15	38.46%
Spindle cells	9	23.08%
Immature ovoid cells	4	10.26%
Pleomorphic	3	7.69%
Immature spindle cells	2	5.13%
Cross-striations	1	2.56%
Homogeneous	1	2.56%
Cytoplasm		0.00%
Eosinophilic Cytoplasm	6	15.38%
Abundant	2	5.13%
Clear	4	10.26%
Scanty	4	10.26%
Eosinophilic cytoplasmic globules/Globoid cytoplasm	2	5.13%
Atypia	1	2.56%
Indistinct	1	2.56%
Nucleus		0.00%
Hyperchromatic	9	23.08%
Elongated	3	7.69%
Pleomorphic	5	12.82%
Eccentric	2	5.13%
Coarse chromatin	2	5.13%
Prominent Nucleoli	2	5.13%
Ovoid	1	2.56%
Round	1	2.56%
Irregular	1	2.56%
Round to oval	1	2.56%
Special cells present		
Rhabdomyoblast /skeletal muscle differentiation	10	25.64%
Multinucleated giant cells	5	12.82%
Cartilaginous differentiation	2	5.13%
Strap cells	2	5.13%
Patterns		
Necrosis	7	17.95%
Myxoid	4	10.26%
Fascicular	5	12.82%
Calcification	2	5.13%
Well-vascularized	3	7.69%
Storiform	2	5.13%
Lobular/alveolar	1	2.56%

Additionally, the most common histological types encountered were ten embryonal, out of which one was an anaplastic variant. Five were alveolar rhabdomyosarcoma. Rest of the histopathology subtypes were not specified.

There were a variety of immunohistochemistry findings, with the most common being desmin in 25 cases (60.97%), followed by myogenin in 19 cases (46.34%). There was a variety of post-operative complications reported, with the most common being recurrence in 15 cases (36.59%). Among the outcomes, 21 (53.85%) died, 13 (33.33%) survived till the last follow-up and the status of five patients is not specified . Among the types of adjuvant therapy used in patients, combined chemoradiotherapy was used in 14 cases (52.63%), radiotherapy only was used in nine cases (15.79%), chemotherapy only was used in six cases (10.53%), and six cases were unspecified (21.05%). There were a variety of chemotherapy drugs used, with the most common being vincristine in 14 cases (42.11%), followed by cyclophosphamide in 10 cases (36.84%) and etoposide in five cases (26.32%). The least common were doxorubicin and pirarubicin, each present in one case (5.26%). The follow-up of 28 patients is mentioned with a mean of 14.39±17.68 months.

## DISCUSSION

### Oncologic Origin:

***a) Genetic Factors in the Development of PIRMS:*** Understanding PIRMS aetiology requires examining RMS origins. RMS, soft tissue sarcomas from primitive mesenchymal tissue, can develop in various locations, including the extremities (ERMS), head/neck (HNRMS), and genitourinary tract (GRMS).**2,33** RMS are also linked to several familial syndromes, such as Li-Fraumeni, Costello, Beckwith-Wiedemann, Neurofibromatosis type 1, DICER1, Gorlin basal cell nevus, and Rubinstein-Taybi syndromes, many of which are associated with germline mutations, including mutations in TP53, NF1, HRAS, and DICER1, increasing the risk of developing embryonal RMS.^33^ Some of these familial syndromes show site-specific RMS development; DICER1 mutations are common in GRMS, while TP53 mutations occur across various sites, including the head/neck, extremities, and genitourinary tract.^2^,^33^ Two cases of PIRMS from our review have been reported with somatic DICER1 mutations, exhibiting similar clinical and pathological features.^7^ Although in a few cases, this correlation suggests a possible oncogenic origin of PIRMS. A case report of a patient with Neurofibromatosis 2, linked to the NF2 gene, later developing RMS suggests a possible role of NF2 mutations in RMS development.^17^ Another patient was diagnosed with hypomelanosis of Ito, a genetic neurocutaneous disorder, and as mentioned previously Neurofibromatosis 1 and Gorlin syndrome have been linked to RMS.^22^,^33^ Thus, despite limited genetic testing in the majority of cases, it is reasonable to infer a genetic component could be contributing to PIRMS development.

***b) Cancer Stem-Cells in Tumour Initiation and Growth of PIRMS:*** The stem cell hypothesis suggests that tumours contain a subset of stem-like cells.^34^ These cells, known as cancer stem cells (CSCs), are slow-cycling and can initiate tumour growth. Unlike most rapidly dividing tumour cells, CSCs are thought to fuel tumour growth and progression.

CSCs are believed to resist chemotherapy and radiation but can regenerate and produce diverse cell types within tumours, driving disease growth and recurrence even after treatment.^35^ Although studied in vitro, CSCs’ role in the tumour microenvironment remains under research. In the context of PIRMS, these CSCs may initiate and sustain tumour growth through continuous proliferation. Furthermore, the aggressive nature of PIRMS tumours can make complete surgical resection difficult, as noted in approximately 41.02% of patients achieving only subtotal resection. 6,11,13,14,15 Additionally, despite aggressive treatment approaches, including surgery, chemotherapy and radiation, PIRMS was recurrent in 36.59% of the patients.^16^,^19^,^21^ The presence of CSCs in PIRMS highlights the need to study their role in the tumour microenvironment, crucial for developing better treatments and improving patient outcomes.

### Demographic patterns of PIRMS:

RMS primarily affects children, accounting for half of pediatric soft tissue sarcomas, though it can also occur in adults. The incidence of RMS among individuals under the age of 20 is approximately 4.5 cases per million.^36^,^37^ The age and gender distribution among different types of RMSs, including ERMS, HNRMS, and GRMS, reveal interesting patterns. ERMS predominantly affects children under the age of 10, with an equal gender distribution.^38^ Conversely, HNRMS tends to occur in slightly older individuals, with a median age at diagnosis of 6.4 years, and a slight excess of males. GRMS shows a bimodal distribution, affecting toddlers aged 2-4 years and adolescents aged 15-19 years.^2^ PIRMS differs demographically, with a higher mean age of 35.91 years in adults and 8.13 years in pediatric cases, along with an almost equal gender distribution. This suggests that PIRMS may have a later onset than other RMSs, which primarily affect children and adolescents. Racially, among 39 patients, 25 were Asian, 11 Caucasian, two Hispanic, and one African-American, indicating a predominance of cases reported in Asian populations. More data is needed to confirm gender predispositions and their prevalence in adults.

**Table-VII:** APPENDIX Joanna Briggs Assessment for Case Reports of Included Articles.

Author	Q1	Q2	Q3	Q4	Q5	Q6	Q7	Q8	Overall Appraisal
Mondrago et al.^6^	Y	Y	Y	Y	Y	Y	U	Y	Included
Vaidya et al.^8^	Y	Y	Y	Y	Y	Y	U	Y	Included
Yoshida et al.^9^	Y	Y	Y	Y	Y	Y	Y	Y	Included
Duncan et al.^10^	N	Y	Y	Y	Y	Y	Y	Y	Included
Ishi et al.^11^	Y	Y	Y	Y	Y	Y	U	Y	Included
Lau et al.^12^	Y	Y	Y	Y	Y	Y	U	Y	Included
Nair et al.^13^	Y	Y	Y	Y	Y	Y	Y	Y	Included
Scull et al.^14^	Y	Y	N	Y	Y	Y	Y	Y	Included
Palta et al.^15^	Y	Y	Y	Y	Y	Y	U	Y	Included
Pirillo et al.^16^	N	Y	Y	Y	Y	Y	Y	Y	Included
Carlson et al.^17^	Y	N	Y	Y	Y	Y	U	Y	Included
Lee et al.^18^	Y	Y	Y	Y	Y	Y	Y	Y	Included
Grebe et al.^19^	Y	Y	Y	U	Y	Y	Y	Y	Included
Guilcher et al.^20^	Y	Y	Y	Y	Y	Y	Y	Y	Included
Arita et al.^21^	Y	Y	Y	Y	Y	Y	U	Y	Included
Mahmasani et al.^22^	Y	Y	Y	Y	Y	Y	Y	Y	Included
Nishikawa et al.^23^	Y	N	Y	Y	Y	Y	U	Y	Included
Pandey et al.^24^	Y	Y	Y	Y	Y	Y	U	Y	Included
Masoudi et al.^26^	Y	N	Y	Y	Y	Y	Y	Y	Included
Jour et al.^27^	Y	Y	Y	U	Y	Y	Y	Y	Included
De Leeuw et al.^28^	Y	Y	Y	Y	Y	Y	Y	Y	Included
Desai et al.^29^	Y	Y	Y	Y	Y	Y	Y	Y	Included
Garvia et al.^30^	Y	Y	Y	Y	Y	Y	U	Y	Included
Zhong et al.^31^	Y	Y	Y	Y	Y	Y	Y	Y	Included
Xie et al.^32^	Y	Y	Y	Y	Y	Y	Y	Y	Included

**Table-VIII:** Joanna Briggs assessment for case series of included article.

Author	Q1	Q2	Q3	Q4	Q5	Q6	Q7	Q8	Q9	Q10	Overall Appraisal
Sakaguchi et al.^7^	Y	N	Y	Y	Y	Y	U	U	Y	Y	Included

**Table-XI:** Joanna Briggs assessment for the cross-sectional study of the included article

Author	Q1	Q2	Q3	Q4	Q5	Q6	Q7	Q8	Overall Appraisal
Zheng et al.^25^	Y	Y	U	Y	Y	Y	Y	Y	Included

### Radiological Characteristics of PIRMS:

The CT findings for PIRMS included haemorrhage, ventriculomegaly as the most commonly observed findings, each occurring in approximately 10.26% of cases respectively.^6^,^7^,^11^,^20^,^22^ These findings suggest the presence of increased intracranial pressure, which are typical manifestations of intracranial tumours. oedema, cystic components, and midline shift were noted in 5.13% of cases, indicating the potential for peritumoral oedema and cystic degeneration within the tumour.^7^,^16^,^17^,^19^ These features can contribute to the clinical presentation of symptoms such as headaches and neurological deficits. Other less common findings included herniation and ventricular rupture.^6^,^7^,^16^ These findings may indicate advanced disease or complications from tumour growth within the confined intracranial space.

MRI is the preferred imaging modality for RMS due to its superior soft-tissue contrast and lack of ionising radiation. However, CT of the abdomen, chest, and pelvis is recommended for metastasis screening.^12^ When comparing the MRI findings of PIRMS to those of other RMSs subtypes such as GRMS, HNRMS, and Parameningeal Rhabdomyosarcoma (PRMS), several similarities and differences can be observed. In PIRMS cases, 51.28% exhibit a hypointense mass on T1-weighted images (T1WI), while a smaller percentage of 5.13% appear iso-intense on T1WI.^7^,^9^,^13^,^16^,^21^ Additionally, 41.03% of cases show a hyperintense mass on T2-weighted images (T2WI), indicating variable signal intensities within the tumour.^7^,^12^,^13^,^16^,^21^ These findings contrast with HNRMSs, which typically appear iso- to hyperintense on T1WI. However, similar to PIRMS, HNRMSs and PRMSs both present as hyperintense on T2WI, likely due to haemorrhage or necrosis.^39^ Approximately 15.38% of PIRMS cases exhibit a cystic component, suggesting areas of necrosis or cystic degeneration, while similar features are present in PRMS.^9^,^16^,^18^,^19^,^39^ Enhancement and mass effect were noted in 25.64% and 15.38% of cases, indicating the presence of tumour enhancement post-contrast administration and the associated compression of adjacent structures, respectively.^7^,^9^,^21^Additionally, the midline shift seen in 10.26% of cases further solidifies this observation, all of which are also seen in other RMSs. 6,10,22,20,39 Furthermore, GRMS exhibits similar MRI characteristics on both T1WI and T2WI compared to other RMSs.^29^ While PIRMS shares some MRI features with other RMS types, differences stem from tumour location, growth pattern, necrosis, and surrounding anatomy.

### Histopathological Profile of PIRMS:

RMS consists of four histopathological subgroups: spindle cell/sclerosing, pleomorphic, alveolar (ARMS), and embryonal (EmRMS).^40^ Among these, EmRMS predominates, constituting about 75% of all RMS cases, with ARMS representing around 20%.^39^ EmRMS demonstrates an equal distribution between the head and neck regions and the genitourinary system, while ARMS is observed in the extremities.^39^,^41^ In our review, ten cases of PIRMS patients were diagnosed with EmRMS, among which one case presented as an anaplastic variant. ARMS was observed in five of the cases, while the classification of the remaining eight cases was undefined. ARMS are aggressive with a poorer prognosis than EmRMS, while EmRMS with diffuse anaplasia may have the worst outcomes among its subtypes.^33^,^41^ EmRMS appears more common in PIRMS and may have a milder course than ARMS. However, aggressive subtypes like EmRMS with diffuse anaplasia highlight the importance of histopathological classification in prognosis and treatment planning.

### Immunohistochemistry and Molecular Techniques in PIRMS Diagnosis:

Positive nuclear staining for both markers is essential for differentiating RMS from other neoplasms and distinguishing its subtypes. Additionally, cytoplasmic vimentin and desmin are seen in undifferentiated cells, while muscle actin and myoglobin indicate differentiated rhabdomyoblasts.^42^,^43^ Desmin, commonly used in diagnosis, shows diffuse staining in EmRMS. In contrast, Myogenin and MyoD1 staining in EmRMS are patchy, while ARMS exhibits strong, diffuse nuclear staining. The differences in myogenin staining patterns help identify subtypes, especially in cases with limited biopsy material. Many ARMS cases in the head and neck region show epithelial marker positivity, further differentiating them from EmRMS.^41^ Recent research also emphasised the significance of immunohistochemical markers like p53, Ki67, and MIB-1 expression as prognostic factors in RMS diagnosis.^42^,^43^

In our review, most PIRMS patients showed desmin positivity (60.97%), indicating consistent muscle differentiation. Myogenin was positive in 19 of 39 cases, reinforcing its role in rhabdomyoblast identification. Muscle and proliferative markers like smooth muscle actin, MyoD1, and Ki-67 were positive in some cases, highlighting PIRMS heterogeneity. p53 and MIB-1 showed lower positivity, reflecting variability in expression and prognostic significance. RMS can be diagnosed even with minimal skeletal muscle protein expression, indicating a failure in terminal differentiation, a key RMS characteristic. However, cellular morphology remains crucial, as myogenic proteins may also appear in other childhood neoplasms like Wilms tumours.^36^ A panel of immunohistochemical markers is essential for accurate PIRMS diagnosis and prognosis.

Recent studies highlight the molecular characterisation of ARMS, focusing on a recurrent chromosomal translocation involving transcription factor-encoding genes PAX3 or PAX7 and FOXO1. These fusion events contribute to metastasis, recurrence, and therapy resistance in ARMS. Although linked to a more aggressive disease, fusion-positive cases show low prognostic significance, suggesting advanced disease but not necessarily a worse outcome. The World Health Organisation Classification of Tumours of the Central Nervous System has outlined key diagnostic criteria for RMS, including the presence of a malignant primitive tumour showing at least focal immunohistochemical evidence of skeletal muscle lineage and the absence of non-rhabdomyosarcomatous components. In diagnostically challenging cases, confirmation of a FOXO1 gene fusion is needed.^43^ FOXO1 gene fusion status was unreported in PIRMS cases in our review. However, its identification could be clinically significant for prognosis and treatment planning.

### Navigating Surgical Approaches in PIRMS:

Due to the lack of definitive CT or MRI findings for RMS and its rarity in the brain, neoadjuvant therapy was not feasible in most cases. Initial surgical excision was performed, followed by histopathological and immunohistochemical analysis for diagnosis. The surgical approach varied based on tumour location, ranging from invasive to non-invasive procedures. This approach highlights a key challenge in RMS cases: achieving complete tumour resection while preserving function and aesthetics amid complex anatomical constraints.^33^ Notably, primary surgical resection is less common in GRMS and HNRMS due to the high risk of severe mutilation.^1^,^33^ Due to PIRMS’ complex anatomical location, complete tumour resection is challenging and was achieved in only 36.84% of cases^9^,^10^,^16^,^19^,^20^,^21^ For HNRMS, some authors recommend an initial excisional biopsy followed by delayed primary excision, while others favour early complex surgery and a multidisciplinary approach. In contrast, ERMS tumours, with more favourable anatomy, benefit from primary surgical excision, leading to higher survival rates and lower recurrence.^38^ It is noteworthy that complete surgical resection followed by adjunctive therapy improves prognosis across all RMS types.^1^,^2^,^44^

### Adjuvant Therapy in PIRMS:

The current RMS treatment protocol follows a multimodal approach, starting with a biopsy unless complete excision is possible, followed by neoadjuvant chemotherapy and radiotherapy, with or without delayed surgery. Its success relies on accurate diagnosis, staging, and comprehensive local and systemic therapy.

### Risk Stratification:

Risk stratification guides treatment selection, optimising therapy while minimising risks and side effects. However, variations in criteria can influence treatment decisions.^36^ Low-risk patients usually have localized, histologically confirmed EmRMS in favourable sites (non-parameningeal, orbit, vagina/uterus), grossly excised EmRMS, or EmRMS limited to the orbit. Conversely, high-risk patients may have lymph node involvement or metastases, histologically confirmed ARMS, or tumours located in unfavourable anatomical sites (bladder, prostate, parameningeal region, or extremities.^36^ While certain risk factors for developing primary intracranial alveolar rhabdomyosarcoma have been identified in the pediatric population—such as age, gender, and inherited syndromes—corresponding risk factors in adults remain undefined, as noted by Desai et al.^29^

### Chemotherapy:

The primary chemotherapy regimen for RMS typically involves an alkylating-based combination of vincristine and actinomycin D at intervals of 6-9 months. In North America, this combination includes cyclophosphamide (VAC), while in Europe, ifosfamide (IVA) is commonly used. A randomised trial found VAC versus IVA as the initial therapy, followed by VAC for all patients, equal in effectiveness.^2^ A recent low-risk EmRMS trial reduced chemotherapy to 24 weeks with lower cyclophosphamide doses, while another group maintained standard vincristine and actinomycin D for 48 weeks with reduced cyclophosphamide. Despite improved three-year survival, failure-free survival remained suboptimal, highlighting relapse risks and the potential need for more aggressive treatment.^36^

In our review, most cases received chemoradiotherapy, while nine had radiotherapy alone and six had chemotherapy alone. Patients treated with chemoradiotherapy showed a better prognosis than those receiving single-modality treatment. Vincristine was the most used chemotherapy drug (42.11% of cases), followed by cyclophosphamide (36.84%). Etoposide, carboplatin, and ifosfamide were commonly used, while cisplatin, actinomycin D, dactinomycin, temozolomide, doxorubicin, and pirarubicin were used in less than 16% of cases. Current evidence indicates that adjuvant chemotherapy remains the most effective treatment strategy for PIRMS. Regimens such as VAC, and combinations including doxorubicin and ifosfamide were associated with survival beyond 12 months, with some cases exceeding five years.^25^ This diversity in chemotherapy underscores the need for individualized treatment regimens to optimise outcomes in PIRMS patients.

### Radiotherapy:

In traditional RMS treatment, radiotherapy after surgery remains key for local control. Modern practice adjusts radiation dosage based on tumour resection to reduce side effects, as radiotherapy increases the risk of chronic health conditions fivefold in survivors.^45^ While radiation is sometimes avoided in young patients, many still receive it due to the aggressive pathology of PIRMS. Higher doses have generally been linked to longer survival, although a clear dose-response relationship has not been confirmed due to lack of data.^25^ ARMS patients treated with chemotherapy and radiotherapy often achieve high control rates, highlighting the disease’s aggressive nature.^45^ Whereas GRMS patients, especially those with vaginal tumors, often respond well to chemotherapy alone, avoiding the need for radiotherapy.^36^,^46^ In our review, patients treated with radiotherapy alone had varied prognoses, making its effectiveness as a standalone treatment uncertain.

### Outcomes and Follow-up:

In our review, the overall survival rate was 33.33%, with 53.85% of patients dying during the follow-up period. Thirteen patients (33.33%) remained alive at the last follow-up, while the survival status of five was not specified. Among treatment modalities, surgery combined with chemoradiotherapy was most frequently employed (52.63%) and was associated with a better prognosis compared to single-modality treatment approaches, suggesting a potential survival advantage with multimodal therapy. Patients treated with surgery and radiotherapy alone (15.79%) or surgery and chemotherapy alone (10.53%) demonstrated comparatively poorer outcomes, while those managed with surgery alone (n=3) had particularly worse prognoses with two deaths and one outcome unknown. These findings are consistent with the data from Zheng et al., in which adjuvant chemotherapy was associated with significantly better overall survival, with a 47.6% reduction in local recurrence risk and a 32.4% reduction in mortality risk; notably, all patients who developed local–regional recurrence declined further treatment and died of the disease during follow-up.^25^ This further supports the potential benefit of integrating a multi-modal approach in the management of PIRMS, although the small sample size limits the strength of these observations.

### Limitations:

It is important to acknowledge the potential limitations of our review. Firstly, the relatively small number of reported cases of PIRMS limits the generalizability of our findings. This scarcity of cases hampers our ability to draw definitive risk factors, conclusions and may introduce biases in our analysis. Additionally, the absence of essential data components, including information on genetic factors, histological subtypes, specifics regarding chemotherapy and radiotherapy regimens, and long-term survival outcomes, further restricts the depth of our analysis. Addressing these limitations in future research endeavors is essential to provide a more comprehensive understanding of PIRMS and improve patient care and outcomes. Despite these limitations, our review serves as a foundational resource for further research and clinical practice in the realm of PIRMS.

## CONCLUSION

This systematic review sheds light on the intricate landscape of PIRMS, offering valuable insights into its aetiology, diagnosis, and management strategies. Through an exploration of potential oncologic origins, including genetic factors and the role of cancer stem cells, we have begun to unravel the complex mechanisms underlying PIRMS development. Furthermore, our review underscores the significance of accurate risk stratification and personalised treatment approaches in optimising patient outcomes. A predilection towards the asian and adult male population is an indication that racial and genetic elements are at play. Despite limitations, PIRMS remains difficult to diagnose preoperatively, with histopathology as the gold standard. However, hyperintense mass on T2WI, hyperintense mass on T1WI and enhancement can aid in diagnosis radiologically. A preoperative biopsy can also help in diagnosis and optimising the management plan. Although neo-adjuvant therapy was not utilised much, no apparent benefit was seen over surgery and adjuvant therapy. Treatment response varies, but adjuvant chemoradiotherapy and gross total resection can provide a survival benefit, highlighting the need for further research.

**Fig.2:**
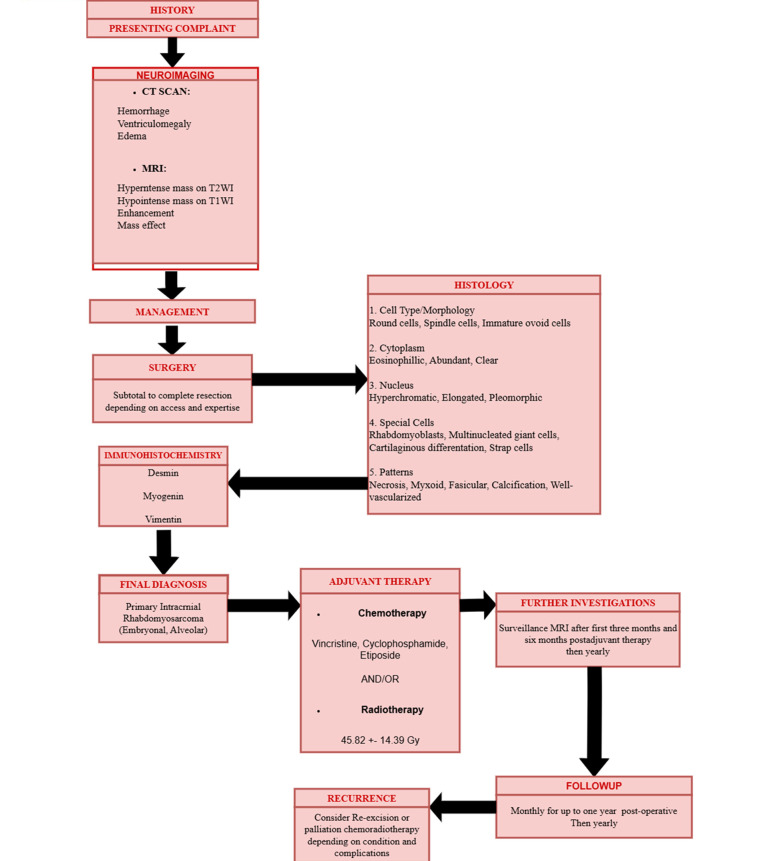
Schematic flowchart for the diagnosis and management of Primary Intracranial Rhabdomyosarcoma.

Fig.2 illustrates a flowchart for clinicians to understand the protocol to follow for PIRMS according to the results of the systematic review.

### Clinical Recommendations:

A preoperative biopsy should be carried out before undergoing definitive resection. After confirmation of primary intracranial rhabdomyosarcoma, where possible, grossly complete resection should be carried out. Where the tumour is large, a possible neo-adjuvant downstage should be considered to render the patient as a candidate for complete resection. As there is a genetic element in RMS, other syndromes associated with RMS should be ruled out and treatment should be planned accordingly. Both combined chemoradiotherapy should be considered as adjuvant therapy for a better clinical response, outweighing risks versus benefits. Post-operative MRI should be carried out, and the patient should be followed for the possibility of recurrence, especially in subtotal resections.

### Author`s Contribution:

**HMQ:** Concept of the study, Critical review of the manuscript and Supervision

**MK:** Data acquisition and interpretation and drafted the manuscript

**AAK:** Data analysis and interpretation and drafted the manuscript and critically reviewed it

**HA and MB:** Data interpretation and drafted the manuscript

**SSHS:** Concept of study and critically reviewed the manuscript

**AB:** Data interpretation and critically reviewed the manuscript.

All the authors have read and approved the final manuscript and are responsible and accountable for the accuracy and integrity of the work.
